# Supplementary material for: Evolutionary trends in Bombella apis CRISPR-Cas systems
Source: mSystems. 2025 Jun 18;10(7):e00166-25. doi: 10.1128/msystems.00166-25 (PMC12282179; doi:10.1128/msystems.00166-25)
Supplement: Supplemental legends — Legends for supplemental tables. [file msystems.00166-25-s0001.rtf]

TABLE S1. Type II-C repeats identified across Bombella genomes.
TABLE S2. Number of shared spacers, gANI, and total mismatches across Bombella genomes.
TABLE S3. Accession numbers for the publicly available genomes used in this study.
TABLE S4. Queen metagenome metadata used in this study and previously published.
TABLE S5. ID of putative viral bins from metagenomic assemblies of the queen microbiome.
